# Plasma Extracellular Vesicle‐Derived miR‐296‐5p is a Maturation‐Dependent Rejuvenation Factor that Downregulates Inflammation and Improves Survival after Sepsis

**DOI:** 10.1002/jev2.70065

**Published:** 2025-04-26

**Authors:** Lun Cai, Parmita Kar, Yutao Liu, Xiaogang Chu, Ashok Sharma, Tae Jin Lee, Ali Arbab, Raghavan Pillai Raju

**Affiliations:** ^1^ Department of Pharmacology and Toxicology, Medical College of Georgia Augusta University Augusta Georgia USA; ^2^ Department of Cell Biology and Anatomy Medical College of Georgia Augusta Georgia USA; ^3^ Center for Biotechnology and Genomic Medicine Medical College of Georgia Augusta Georgia USA; ^4^ Georgia Cancer Center Medical College of Georgia Augusta Georgia USA; ^5^ Charlie Norwood VA Medical Center Augusta Georgia USA

## Abstract

There is a progressive decline in physiological function with age, and aging is associated with increased susceptibility to injury and infection. However, several reports have indicated that the agility of youth is characterized by transferable rejuvenating molecular factors, as was observed previously in heterochronic parabiosis experiments. These experiments demonstrated a rejuvenating effect of young blood in old animals. There have been several efforts to characterize these youthful or maturation‐associated factors in the young blood. In this report, we demonstrate the resilience of young mice, at or before puberty, to polymicrobial sepsis and show an age‐dependent effect of small extracellular vesicles (EVs) from plasma on the outcome following sepsis. The EVs from the young mice were cytoprotective, anti‐inflammatory, and reduced cellular senescence markers. MicroRNA sequencing of the EVs showed an age‐associated signature and identified miR‐296‐5p and miR‐541‐5p to progressively reduce their levels in the blood plasma with increasing age. We further show that the levels of these miRNAs decline with age in multiple organs. The miRNAs miR‐296‐5p and miR‐541‐5p showed a reparatory effect in an in vitro wound healing model and the miR‐296‐5p, when given intraperitoneally, reduced mortality in the mouse model of sepsis. In summary, our studies demonstrate that EVs from very young mice have a reparative effect on sepsis, and the reparative factors are likely maturation‐dependent. Our observation that miR‐296‐5p and miR‐541‐5p are plasma EV constituents that significantly reduce with age and can reduce inflammation suggests a therapeutic potential for these miRNAs in inflammation and age‐associated diseases.

**Abbreviations**
ccf‐mtDNA: circulating cell‐free mitochondrial DNA
CLP: caecal ligation and puncture
COX‐3: cyclooxygenase‐3
CXCL4: chemokine (CXC motif) ligand 4
DAMPs: damage‐associated molecular patterns
EVs: exosomes
GDF11: growth differentiation factor 11
GST: glutathione S‐transferase
GSTM2: Glutathione S‐transferase Mu 2
HI: hemorrhagic shock injury
LDH: lactate dehydrogenase
MEF: mouse embryonic fibroblasts
MSC: mesenchymal stem cell
ND2: NADH dehydrogenase subunit 2
ND3: NADH dehydrogenase subunit3
ND5: NADH dehydrogenase subunit5
PDGFRβ: platelet‐derived growth factor receptor beta
PF4: platelet‐derived factor 4
PMVEC: pulmonary microvascular vascular endothelial cells
SPECT: single‐photon emission computed tomography
VEGF: vascular endothelial growth factor
VEGFR: vascular endothelial growth factor receptor

## 1 Introduction

Aging is an inevitable biological process and is associated with a progressive decline in physiological function. Though the discovery of methods to extend longevity is much sought after, finding methods for healthy aging may be a more attainable goal. Several agents that modulate autophagy, mitochondrial function, senescence, and antioxidant responses have been shown to promote healthy aging, and their effects may be attributed to the functionally interrelated nature of these biological processes and functions (Blagosklonny 2022; Baur and Sinclair 2006; Shade 2020). There have also been several attempts to identify rejuvenating endogenous factors; these include metabolic regulators and various growth factors native to the being (Rochette et al. 2023).

The resilience of the young organism to injury and infection is well‐documented (Rogers and Lucchesi 2014; Khalil et al. 2021; Chu et al. 2022). Further, heterochronic parabiosis experiments have demonstrated that young blood can rejuvenate aging systems (Conboy et al. 2013). However, the factors in young blood that are responsible for the rejuvenating effect, or those in the old animal that trigger organismal aging, remain unknown or, at best, speculative (Chu and Raju 2023). For example, it was found that levels of growth differentiation factor 11(GDF11) were reduced with age in mice, and its restoration reduced experimental cardiac hypertrophy and improved muscle injury in the old mice (Sinha et al. 2014). In another study, small extracellular vesicles (EVs) isolated from young human donor fibroblasts were found to reduce senescence markers and oxidative stress in fibroblasts derived from old donors (Fafian‐Labora et al. 2020). The young EVs were found to have high levels of glutathione S‐transferase Mu 2 (GSTM2) activity, which led to the conclusion that intrinsic glutathione S‐transferase (GST) activity in EVs mediated the rejuvenation of fibroblasts from old donors (Fafian‐Labora et al. 2020). Further reinforcing the role of circulatory factors in potentiating the aged organ systems, it was found that EVs isolated from young mice rejuvenated aged cell bioenergetics and skeletal muscle regeneration (Sahu et al. 2021). They also found the levels of KLOTHO in the young EVs declining with donor age, confirming previous studies that showed a diminution of circulating KLOTHO levels with aging and tissue rejuvenation following its upregulation (Sahu et al. 2021; Buchanan et al. 2020). Similarly, multiple laboratories have reported the rejuvenating effect of platelet‐derived factor 4 (PF4), also known as CXCL4 (Schroer et al. 2023; Leiter et al. 2023; Park et al. 2023). Nevertheless, it is unclear whether the accumulation of “pro‐aging” factors, the diminution of “pro‐youthful” factors or a combination of both, promotes the aging phenotype (Laviano 2014). We recently reported an age‐associated worsening of outcomes following acute injury using a mouse model of haemorrhagic shock injury (HI), wherein the best outcome after HI was in mice at pubertal age compared to the older ones (Chu et al. 2022). We also showed that EVs isolated from these young mice were more potent in prolonging life in old mice after HI (Chu et al. 2022).

Sepsis is a life‐threatening inflammatory disease in response to infection with an age‐associated increase in mortality (Martin et al. 2006; Michels et al. 2022). Although patients older than 65 years (elderly) accounted for only 12% of the US population, 64.9% of the sepsis cases were in this age group, with similar data reported from other countries (Martin et al. 2006). Using a well‐recognized mouse model of polymicrobial sepsis, developed by caecal ligation and puncture (CLP) (Cai et al. 2022; Lavarti et al. 2024), we addressed the age‐associated difference in mortality following sepsis. In this study, we found that EVs from the plasma of young mice significantly reduced mortality compared to EVs isolated from older mice. The cargo of plasma EVs contains a number of molecular species including miRNAs (Alibhai et al. 2020; Xu et al. 2022; Garcia‐Martin et al. 2022; Liu and Halushka 2024). miRNAs are endogenous small non‐coding RNAs that can bind mRNA targets and regulate gene expression by inhibiting translation or destroying the target mRNA. Although several studies indicate the role of EVs in long‐distance communication by delivering the cargo to other cells, some reports suggest such deliveries are rare or the deliveries take place by kiss‐and‐run interactions (Morris and Witwer 2022; Albanese et al. 2021). Several miRNAs have been found to be dysregulated in sepsis or tested as potential therapeutic agents (Dragomir et al. 2023; Benz et al. 2016; Zheng et al. 2023). We performed microRNA profiling of the plasma‐derived EVs and demonstrated a sharp decline in the level of two miRNAs in the EVs with increasing age. The two miRNAs, miR‐296‐5p and miR‐541‐5p, suppressed LPS‐induced inflammation, and treatment of mice with miR‐296‐5p reduced mortality following sepsis. We report that miR‐296‐5p and miR‐541‐5p are maturation‐dependent factors with a potential therapeutic effect.

## 2 Materials and Methods

### 2.1 Animals

All animal care and use procedures were performed according to the protocol approved by the Institutional Animal Care and Use Committee of Augusta University and were in compliance with the “Guide for the Care and Use of Laboratory Animals” published by the National Institutes of Health. Male C57BL/6 mice of ages 5–6 weeks, 12–16 weeks, 12 months and 23–26 months were used in this study. The mice were purchased from Charles River Laboratory or through the National Institute on Aging and housed in the Augusta University vivarium.

### 2.2 Mouse Model of Polymicrobial Sepsis

As described previously, polymicrobial sepsis was induced by CLP (Cai et al. 2022). In brief, mice were anesthetized with isoflurane (2.5%), the cecum was ligated at ∼40% length from the distal pole, punctured through with a 22‐gauge needle, and gently squeezed to take out a small amount of faecal material. The abdominal incision was closed in two layers. The sham‐operated mice underwent general anaesthesia and laparotomy but not CLP. Carprofen was administrated subcutaneously for pain control immediately before and every 12 h for 24 h after surgery. After the surgery, prewarmed saline (4 mL/kg body weight) was injected subcutaneously. Sepsis score was monitored as described in Garcia‐Martin et al. (2022).

### 2.3 EV Isolation and Characterization

EV donors were male mice, 5–6 weeks, 14–16 weeks, 12 months and 23–26 months old. Blood was collected with a heparin‐wet tuberculin syringe and plasma EVs were isolated by column chromatography using Extracellular Vesicle Size Exclusion Chromatography Columns (STEMCELL technologies, Cambridge, MA, USA). Briefly, plasma collected was diluted with an equal volume of Ca^2+^/Mg^2+^‐free Dulbecco's PBS (D‐PBS) and then centrifuged at 300 *g* for 10 min to remove cell debris and protein aggregates. The supernatant was centrifuged at 1200 *g* for 20 min, followed by 10,000 *g* for 30 min. The supernatant was loaded to the top of the column and 100 µL fractions were collected and quantified using a nano tracking system, Particle Metrix (ZetaView, Ammersee Germany), and 9–14 fractions were found to be consistently enriched for EVs. EV proteins were measured using Micro BCA Protein Assay Kit (Thermo Scientific). Immuno‐electron microscopy was done at the histology core to detect exosomal surface markers, CD9 and CD63.

### 2.4 In Vivo Treatments

To determine the effect of plasma factors from mice of varying ages on sepsis outcome, plasma EVs (10^8^ particles/dose) were injected into recipient mice at 2, 24, and 48 h post‐CLP surgery. The mice were scored for sepsis daily (Cai et al. 2022; Shrum et al. 2014). To determine the effect of miR‐296‐5p, the miRNA mimic or negative control miRNA was administered 24 h before and at 2 h after the CLP surgery intraperitoneally (5 ug/kg) as described previously (Dragomir et al. 2023). In vivo‐jetPEI transfection reagent was used as delivery carrier, the N/P (Nucleotide/PEI) ratio was 6. The transfection mix was incubated at room temperature for 15 min prior to injection. The recipient mice (12–14 weeks) were monitored for sepsis severity and survival.

### 2.5 SPECT/CT Imaging

Single‐photon emission computed tomography (SPECT) images were acquired on a NanoScan (Mediso, Budapest, Hungary) using Indium‐111 trapped EVs. EVs were injected through the tail vein and the images were acquired after 2 h to assess tissue‐distribution of the EVs. Multiplanar SPECT‐CT images were reconstructed for display using manufacturer‐supplied software (Nucline, Mediso). All SPECT images were formatted in Analyze format and multiplanar reconstruction.

### 2.6 RNA and DNA Isolation and miRNA Sequencing

Total RNA in tissues was isolated using TRIzol reagent according to the manufacturer's protocols (Thermal Fisher, Carlsbad, CA, USA; 4478545) (Kar et al. 2024). cfDNA was isolated using Plasma/Serum Cell‐Free Circulating DNA Purification Micro Kit (NORGEN, Cat.55500). Total miRNA was extracted using the PureLink total miRNA Isolation Kit (Invitrogen, Carlsbad, CA, USA; K157001). cDNA from total RNA was synthesized using ImProm‐II Reverse Transcription System (Promega, Madison, WI, USA). cDNA templates were prepared from EV miRNA using Advanced miRNA cDNA Synthesis Kit (TaqMan, A28007). Total RNA in EVs was extracted using the Total Exosome RNA Isolation Kit (Invitrogen, 4478545). RNA quality was assessed using the Agilent 2100 Bioanalyzer. miRNA Library was prepared by using Illumina TruSeq stranded mRNA kit and sequencing was carried out at Novogene (Durham, NC, USA).

#### 2.6.1 Bioinformatics and Statistical Analysis

The raw sequencing reads were analysed with bioinformatics analysis pipeline using FastQC for quality control, Cutadapt for adapter trimming, and STAR for alignment and mm10 mouse genome for references. Differential expression analyses were performed on the miRNA count using DESeq2 package in R. The *p* values were adjusted with false discovery rate, and significance threshold of 0.05 was used.

### 2.7 Cell Culture and Transient Transfection of miRNA Mimics

The primary mouse embryonic fibroblast (MEF) cells were generated from mouse embryos at 12–13 days (Tan and Lei 2019). MEF cells were cultured in DMEM (Dulbecco′s Modified Eagle′s Medium, Gibco 11965‐092) supplemented with 10% foetal bovine serum and penicillin/streptomycin. 0.5 × 10^5^ MEF cells were seeded in 12‐well plates 18–24 h prior to transfection. Synthetic miR‐296‐5p and miR‐541‐5p mimics (from Qiagen) were transfected into cells following the Lipofectamine RNAiMAX Transfection protocol (Invitrogen 13778100). Twenty‐four hours later, cells were treated with lipopolysaccharide (LPS) (10 ng/mL) and cultured for an additional 24 h. MEF cells were cotreated with EV from young, mature and aged mice and either LPS or doxorubicin. LPS‐treated cells were washed twice with PBS and lysed in TRIzol reagent (Invitrogen, #1196018). Doxorubicin (250 nM)‐treated cells were collected and tested for senescence markers p16 and p21 by flow cytometry.

### 2.8 Reverse Transcription‐Real Time Polymerase Chain Reaction (RT‐PCR)

RT‐PCR was performed using standard procedures using real‐time PCR. The expression levels were normalized to β actin. qPCR was performed using a real‐time PCR instrument (Stratagene Mx3000p; Agilent Technologies) with SYBR Advantage qPCR mix. The thermocycling conditions were as follows: initial denaturation for 30 s at 95°C, followed by 40 cycles of 10 s at 95°C and 30 s at 60°C. Relative mRNA expression was calculated using the 2^−ΔΔCq^ method.

### 2.9 LDH Cytotoxicity Assay

MEF cells were plated onto 96‐well plates at a density of ∼9000 cells/well. The next day, the cells were treated with 10^8^ EV particles. The cells were incubated with EVs for 24 h. After incubation, the cells were washed with PBS and treated with LPS (100 ng/mL) for 24 h. After treatment, cell death was evaluated using a CyQUANTTM LDH Cytotoxicity Assay Kit (Invitrogen, Waltham, MA, USA). Cytotoxicity was calculated using the formula: [(EV‐treated LDH activity‐spontaneous LDH activity)/(maximum LDH activity‐spontaneous LDH activity)] × 100.

### 2.10 Flow Cytometry

MEF cells were treated with doxorubicin (250 nM) or vehicle, incubated with EVs, and collected for flow cytometry. To evaluate senescence in MEF cells by flow cytometry, cells were incubated with the following antibodies: p16‐AF647 and p21‐AF488 (Abcam), after treatment with fixation/permeabilization solution (BioLegend, San Diego, CA). EV preparations were stained with 0.5 µg of CD63‐APC (143905, Biolegend) and/or CD9‐FITC (124807, Biolegend) in filtered PBS. The isotype controls, rat IgG2a‐APC and rat IgG2a‐FITC, were included to test for non‐specific binding. All samples were incubated for 30 min at room temperature, protected from light. Log scales were applied on all channels during the acquisition. The samples were analysed using Acea NovoCyte Quanteon (Beckton Dickinson) flow cytometer, and the data were analysed using FlowJo version 10.8.0.

### 2.11 In Vitro Scratch Assay

Rat Pulmonary Microvascular Vascular Endothelial cells (PMVEC) were seeded in a 12‐well plate at a density of 1 × 10^5^ cells/well and cultured until they reached 80% of confluence. Before scratching the cell monolayer, the base of the wells was marked with horizontal reference lines (two parallel lines approx. 0.3 cm apart with a fine tip marker) to ensure uniform scratching and to obtain the same field for each image acquisition time point. The cell monolayer was then scratched with a 1000 µL sterile pipette tip using the pointed end. The detached cells were then washed away with PBS (1X). One millilitre of complete medium (DMEM + 10% FBS + 1X Pen‐Strep, Gibco) with EVs was then added to the cells. To test the effect of miRNA mimics (miR‐296‐5p and miR‐541–5p), the PMVECs were transfected with respective miRNAs 24 h prior to scratching.

Next, the effect of antagomirs was assessed by first incubating the EVs from young animals with respective antagomirs and Lipofectamine for 90 min at room temperature and then added to the cells. As a transfection positive control, EVs isolated from the plasma of young mice were transfected with a non‐targeting miRNA mimic labelled with Cy3 (150 nM, Catalogue #CP‐004500‐01‐05, Horizon Biosciences) using lipofectamine and incubated with PMVECs. The nucleus was stained with Hoechst. We captured micrographs of scratched regions of interest using an Echo‐revolve inverted microscope on a 4X objective at 0, 24 and 48 h post‐scratch. From each well at least 2–3 pictures were captured for the complete scratch area over the horizontal diameter of the well. In the case of a non‐uniform pattern of cell growth in the scratch, multiple images were taken for evaluation. The images from the corners of the well were omitted from the analysis since these images contained the blunt ends of the scratches and were highly variable and inconsistent due to the increased cell density. The total scratch area for each of the four technical replicates was obtained by combining the scratch areas of 2–3 fields of view taken per replicate. The MRI Wound Healing Tool was used to analyze scratch assays as a plugin to Image‐J software (Github).

### 2.12 Biodistribution of miRNA in Mice

To assess the tissue distribution of i.p. injected miRNA in mice, we used a transfection control, that is, a non‐targeting miRNA labelled with Cy3 (5 µg/kg, Horizon Biosciences) in in vivo‐jetPEI transfection reagent. The control mice were injected with vehicle. Three hours after the injection, the mice were euthanized, the thoracic and abdominal cavity were opened, and organs were dissected out and rinsed with PBS. For imaging the organs, we used a whole‐mount immunofluorescence technique with single‐photon excitation, in a Nikon N‐Sparc confocal microscope and captured intrinsic autofluorescence images excited with 488 nm (green) along with target florescence images at 546 nm (red) to provide structural and morphological information. The heart and kidney were sliced longitudinally into half and placed on an imaging dish (Eppendorf) with inside of the tissue facing toward the base of the dish. A small piece of the intestine was cut open and imaged with the inside of the tissue facing toward the base of the dish. Spleen was imaged as it is, and a 2–3 mm thin slice of liver was also imaged. All the slices were imaged at 20X magnification. The Cy3 fluorescence in plasma was quantitated using a fluorimeter (Biotek, Boston).

### 2.13 Statistical Analysis

Statistical analyses were performed using GraphPad Prism. The differences between the two groups were assessed by two‐tailed *t*‐tests. Multiple group comparisons were made by one‐way ANOVA. A two‐way ANOVA was used for the analysis of the results of the scratch assay. *p* < 0.05 was considered significant.

## 3 Results

### 3.1 Age‐Associated Survival After CLP

**FIGURE 1:**
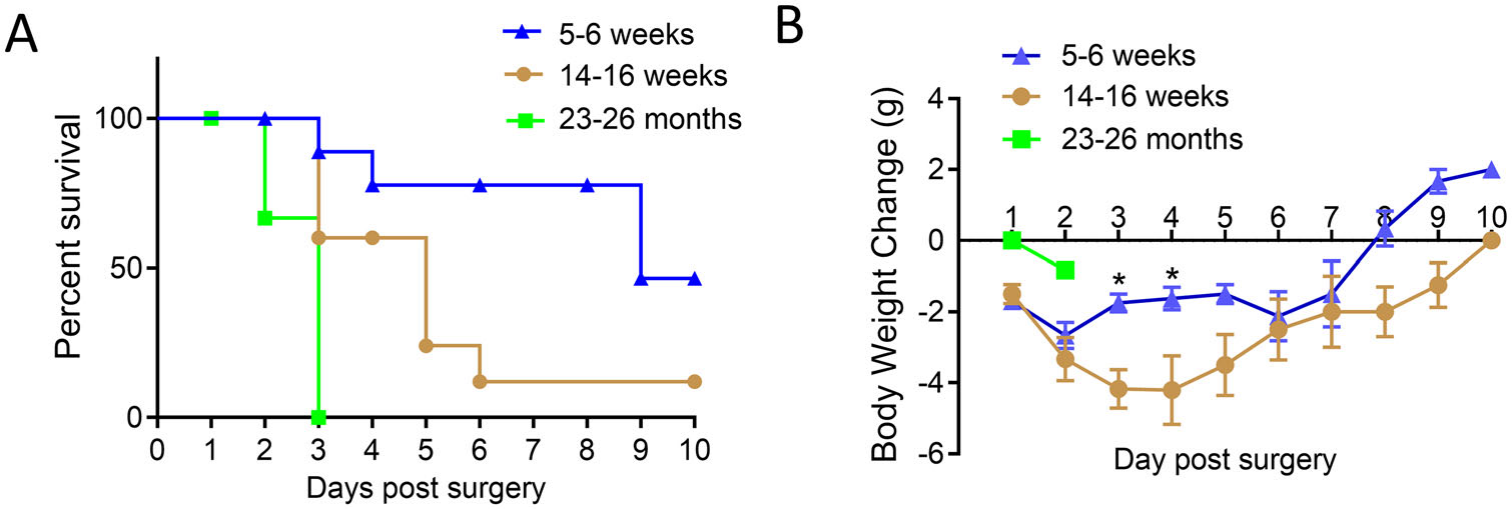
Effect of age on survival after CLP‐induced sepsis. (A) 10‐day survival of mice of three different age groups, following CLP‐induced sepsis, represented by Kaplan–Meier survival curves, show a significantly reduced mortality rate in young mice (5–6 weeks) compared to mature (14–16 weeks) or aged (23–26 months) mice. Young versus mature, *p* = 0.0348; mature versus aged, *p* = 0.0025; young versus aged, *p* = 0.0002. (B) Body weight data *p < 0.05 compared to the mature group (*n* = 10/group).

The effect of age on the survival of mice following CLP surgery was assessed using mice of three different age groups: young (5–6 weeks), mature (14–16 weeks) and aged (23–26 months) (Figure 1A,B). The Kaplan–Meier survival curves for each of the three age groups subjected to CLP showed a progressive increase in mortality with age, demonstrating a strong influence of age on the outcome following sepsis in this experimental model. Although a majority of the mice (80%) in the mature group died within 6 days after CLP, all mice in the aged group died within 3 days (Figure 1A). The 10‐day survival rate among the younger mice was significantly higher than in the mature (*p* = 0.0375) or the aged mice (*p* = 0.0003). The decrease in body weight of the surviving mice in the younger group also remained less than that of mature mice, further demonstrating that young mice were more tolerant to CLP‐induced sepsis.

### 3.2 Plasma EV Size and Number, and mt‐DNA Levels Vary With Age

**FIGURE 2:**
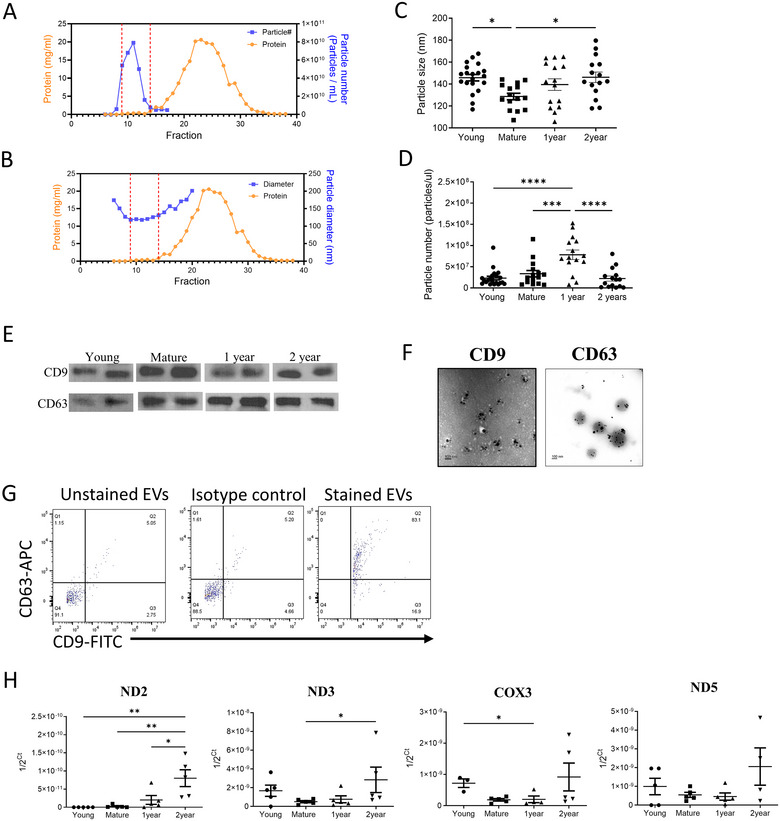
Characterization of plasma EVs. (A, B) Representative elution profile with protein concentration, particle number, and particle diameter of EVs isolated from mouse plasma. The EVs were isolated using size exclusion chromatography as described in Methods. Eluted fractions 9–14 were pooled and used. (C, D) Age‐associated change in EV particle size and number. (E) Western blot analysis of exosome markers in EVs isolated from four different age groups. (F) Representative immuno‐transmission electron microscopy images demonstrating CD9 and CD63 surface markers on the EVs. (G) CD9 and CD63 expression on plasma EVs using flow cytometry. EVs were tested with CD63‐APC, CD9‐FITC and isotype matching antibodies. (H) Circulating cell‐free mitochondrial DNA (ccf‐mtDNA) levels in the plasma EVs changed with age. 10^7^ particles per sample were used to isolate the ccf‐mtDNA (see methods for details). Representative of two independent experiments. *n* = 5/group. Statistical significance was defined as ****p < 0.0001; ***p < 0.001; **p < 0.01; *p < 0.05 for data in respective panels.

The EVs were isolated from the blood plasma using size exclusion chromatography and were characterized by a nanoparticle tracking analyzer (NTA) for count and size, western blot, and flow cytometry for cell surface markers, and transmission electron microscopy for size and specificity. As shown in Figure 2A,B, fractions 9–14 had the most EVs with the least plasma protein content. These fractions were pooled together prior to use. The diameter of the isolated EVs was in the range of 100–200 nm, and the concentration was ∼2–8 × 10^10^ particles/mL/fraction (Figure 2A,B). The average size of EVs from the young and aged mice was significantly higher than those isolated from the mature mice (Figure 2C). However, the number of EVs per microliter from one‐year‐old mice was higher than that in other groups (Figure 2D). In addition, CD9 and CD63, the exosome protein markers, were observed in the protein fraction of EVs isolated from all four age groups by Western blot (Figure 2E). Transmission electron microscopy was used to further confirm the size, morphology and markers of the EVs (Figure 2F). Flow cytometry further confirmed most of the EVs to be CD9 positive and CD63 positive (Figure 2G) with 99% EVs CD9^+^ and 83% CD63^+^. The isolated EV fraction may contain not only exosomes, but also microvesicles, apoptotic bodies and other vesicle types as the markers used for characterization, CD9 and CD63, are not solely exosome‐specific markers but also expressed on, for example, microvesicles budded from the plasma membrane. However, since the EV fraction we selected are in the size range of 100–200 nm, this fraction should be enriched for exosomes.

Based on the prevailing hypothesis that there is an age‐associated increase in circulating cell‐free mitochondrial DNA (ccf‐mtDNA), we tested whether there is a change in the level of mtDNA in the EVs with aging. ccf‐mtDNA fragments are DAMPS and were demonstrated to cause inflammation and tissue damage (Riley and Tait 2020). We amplified four different gene segments on mitochondrial DNA, ND2, ND3, ND5 and COX3, from 10^7^ EV particles and found a significantly progressive increase in ND2 with age (Figure 2H). mtDNA levels were highest in the EVs isolated from the two‐year‐old mice, when compared to all other age groups.

### 3.3 Influence of Donor Age on EV‐mediated Effect on Survival in Mice With Sepsis

**FIGURE 3:**
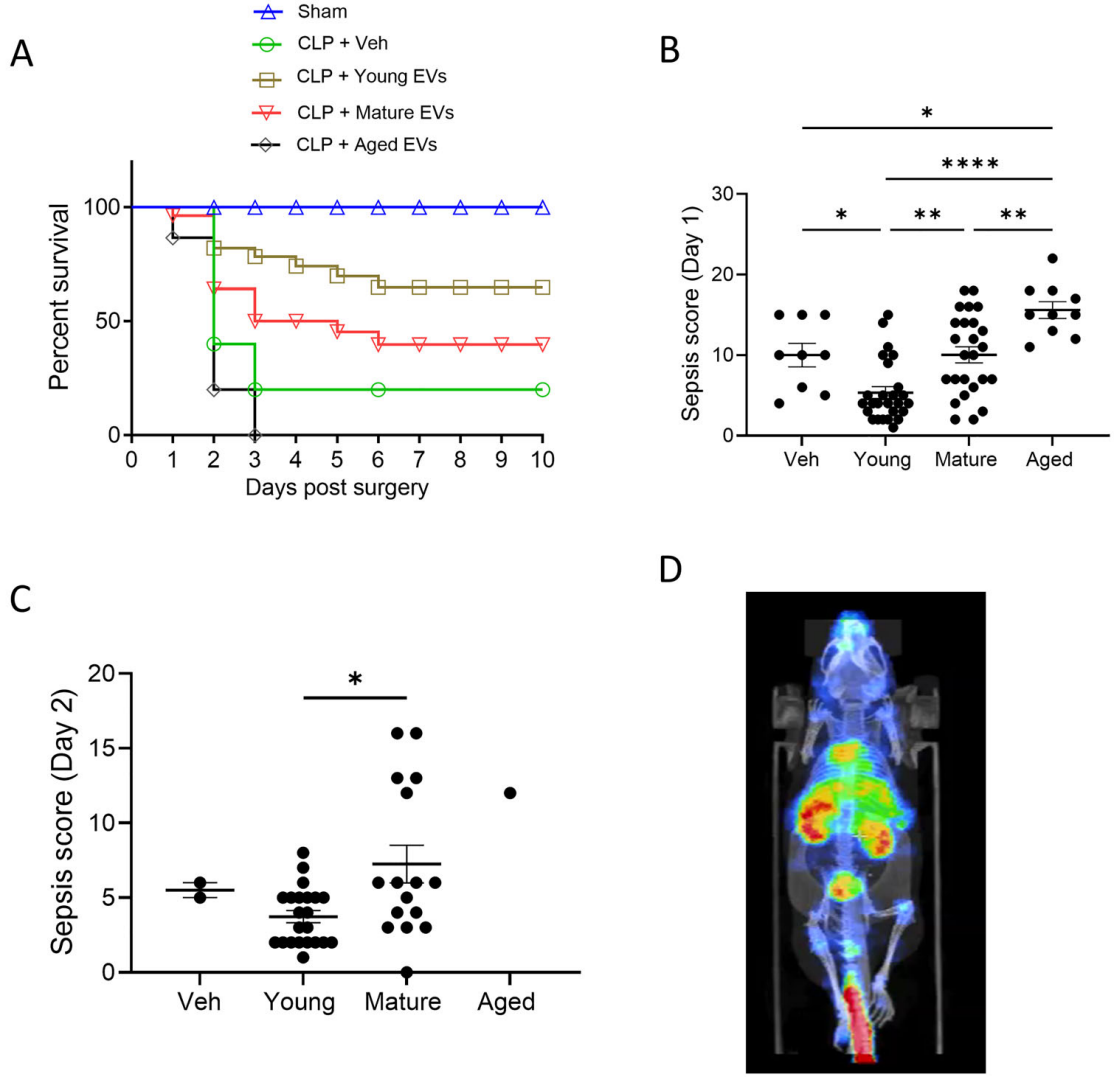
Plasma EVs from young mice reduce mortality following CLP‐induced sepsis. (A) Plasma EVs isolated from young, mature or aged mice were administered (10^8^ particles/dose) to mature mice subjected to CLP or sham surgery at 2, 24 and 48 h after the surgery. Ten‐day survival following CLP‐induced sepsis in each group is represented by Kaplan–Meier curves, and shows the most significant survival in mice that received young EVs. (B, C) Sepsis score on Days 1 (B) and 2 (C) after EVs treatment following sepsis. The data are a composite of three independent experiments. Veh. (*n* = 18), Young (*n* = 26), Mature (*n* = 26) and Aged (*n* = 18). Statistical significance was defined as *p* < 0.05. ****p < 0.0001; ***p < 0.001; **p < 0.01; *p < 0.05. (D) SPECT/CT imaging shows EVs administered through the tail vein homing to multiple internal organs (representative figure).

The EV cargo contains diverse biomolecules derived from various cell types in the organism and have been observed to have a cytoprotective effect for plasma and MSC‐derived EVs in experimental animals (Wiklander et al. 2019). Therefore, we tested whether EV donor age influences the mice's survival outcomes after CLP surgery. The recipient mice received 10^8^ particles/dose of EVs through the tail vein at 2, 24 and 48 h after CLP. In the group of mice that received young EVs, 68% survived, and their 10‐day survival rate was significantly higher in comparison with the vehicle (20%; *p* = 0.0009), mature EV (43%; *p* = 0.045) or aged EV group (0%; <0.0001) (Figure 3A). For a quantitative assessment of sepsis severity, we used a previously established 24‐point sepsis scoring method, as detailed in the methods section (Cai et al. 2022). This enabled us to evaluate the sepsis severity at different time points by monitoring spontaneous activity, response to touch and auditory stimuli, posture, respiration rate, quality and appearance (Figure 3B,C). At 24 h after the surgery, the CLP mice receiving young EVs had lower sepsis scores than those receiving mature EVs, aged EVs, or the vehicle. On Day 2, the composite sepsis severity score in the group that received young EVs continued to be lower than the recipients of mature EVs, while most of the mice that received aged EVs or vehicle did not survive beyond 2 days (Figure 3C). Figure 3D illustrates the propensity of IV‐injected EVs to home to most of the internal organs.

### 3.4 EVs From Young Mice Protect Cells From Inflammation, Senescence and Death

**FIGURE 4:**
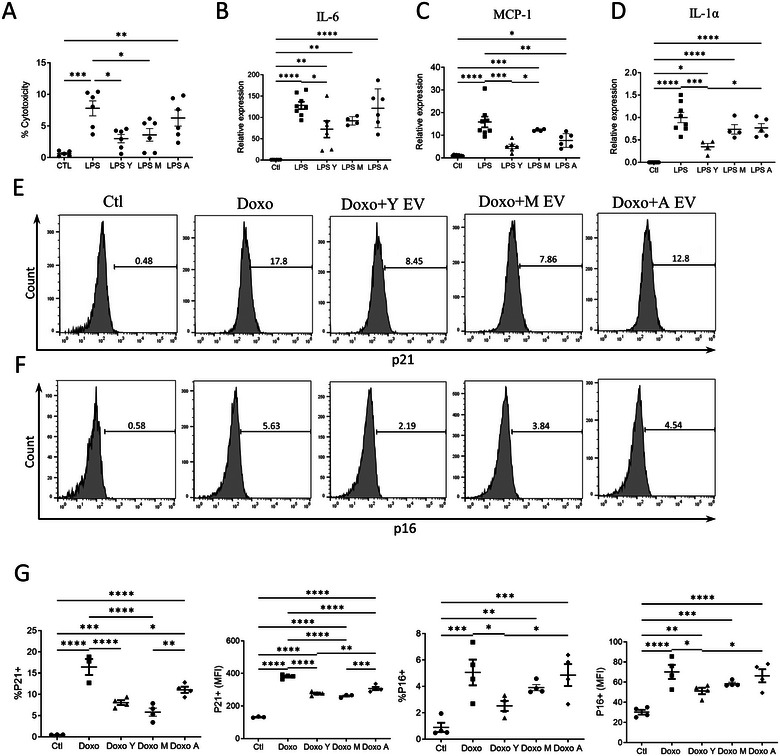
Age‐dependent effect of plasma EVs on inflammation and senescence. (A) Plasma EVs isolated from mice of three different ages were incubated with MEFs challenged with LPS for 24 h and the percentage of total LDH released in the supernatant was measured. Each age group was assayed in six replicates. Young and mature EVs significantly reduced cell death. (B–D) LPS‐treated MEF cells were cultured with EVs, and the expression of inflammatory markers was measured after 24 h by real‐time PCR. Fold induction for each gene was normalized to vehicle control. (E–G) Flow cytometry analysis of the expression of p16 and p21 in MEF cells. Doxorubicin (Doxo)‐treated cells were co‐cultured with EVs for 24 h and tested for the expression of p16 and p21 by flow cytometry.  Results shown as mean ± SEM (*n* = 4–6). Representative of two independent experiments. Statistical significance (one‐way ANOVA) is expressed as *p < 0.05; **p < 0.01; ***p < 0.001; ****p < 0.0001.

The age‐associated propensity to inflammation, cellular senescence and cell death is well described. Sepsis also alters the systemic and tissue microenvironment, triggering immune dysregulation and cell death. To determine whether young EVs confer protection from cytotoxicity and inflammation, we treated MEF cells with LPS and co‐cultured them in the presence or absence of EVs isolated from the young, mature or aged mice. The cytotoxicity, as measured by LDH release, was significantly reduced when the cells were treated with young EVs (Figure 4A). Gene expression of inflammatory markers *Il‐6, Il‐1α*, and *Mcp‐1* was also reduced in the MEF treated with young EVs (Figure 4B–D). Next, we induced senescence in MEF cells by treating them with doxorubicin and tested the effect of EVs. Although doxorubicin treatment increased p21 expression in the MEF cells, the cells co‐treated with young EVs demonstrated downregulation of both p21 and p16, as evidenced by the reduced number and mean fluorescence intensity of p21 and p16 positive cells by flow cytometry (Figure 4E–G).

### 3.5 Age‐Associated Changes in miRNA Profile in the EVs

**FIGURE 5:**
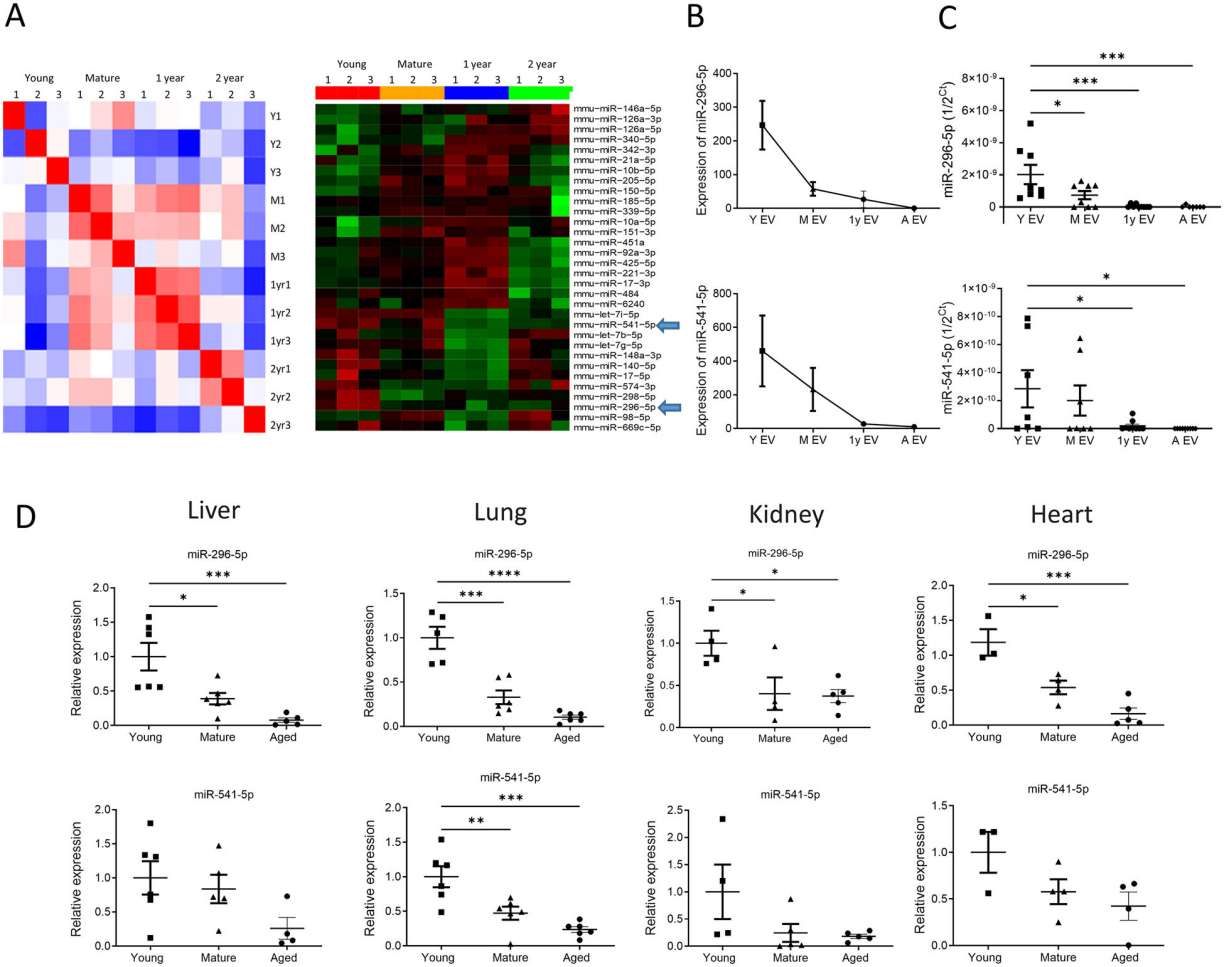
Age‐associated changes in miRNAs in the cargo of plasmas EVs. (A) Left: Sample correlation and cluster analysis of differentially expressed miRNAs in plasma EVs with age (Y = Young, M = Mature). Right: Heatmap of 32 differentially expressed miRNAs which are significant for at least one age against the young group. The two miRNAs (miR‐296‐5p and miR‐541‐5p) that showed a progressive decline in their levels with age are shown by arrows. (B) The graphical representation of miR‐expression data as obtained from miRNA sequencing (left) and miRNA levels in the respective EVs after real‐time PCR amplification (right). (A EV= EV from aged mice) (C) Validation of miR‐296‐5p and miR‐541‐5p levels in plasma EVs from each of the age groups. (D) miR‐296‐5p and miR‐541‐5p expression levels in mouse liver, lung, kidney and heart demonstrate significant reduction with age. Data expressed as mean ± SEM; Statistical significance (one‐way ANOVA) was defined as ****p < 0.0001; ***p < 0.001; **p < 0.01; *p < 0.05.

One of the constituents of EVs is miRNA, and by miRNA sequencing, we profiled the cargo in the plasma EVs derived from mice of four different age groups, ranging from 5 weeks to 2 years. The donor age of the EVs correlated well with the miRNA expression pattern, suggesting an age‐associated miRNA signature in the plasma‐derived EVs (Figure 5A). Our experiment identified 499 miRNAs in the cargo of plasma EVs derived from mice of at least one age group. The expression level of 32 miRNAs showed a significant difference in one or more age groups compared to the levels of respective miRNAs from the young EV group. Figure 5A (right panel) shows these miRNAs in a heatmap. When we looked for miRNAs that showed a progressive decline or increase with age, we found levels of two miRNAs, miR‐296‐5p and miR‐541‐5p, to be sharply declining with age (Figure 5A, right panel). This result was further confirmed by PCR amplification of both miRNAs from the respective EVs; the results showed a consistent decrease with progressive aging (Figure 5B).

We next tested age and tissue‐specific expression of the miRNAs, miR‐296‐5p and miR‐541‐5p, in the liver, lung, kidney and heart of young, mature and aged mice to determine their age‐ and tissue‐dependent changes. The expressions of both miR‐296‐5p and miR‐541‐5p were highest in the young mice, compared to the mature or the aged mice (Figure 5C). The progressively reducing levels of the two miRNAs suggest a relationship between the aging process in critical tissues and these two miRNAs.

### 3.6 The Effect of miR‐296‐5p and miR‐541‐5p on Inflammation

**FIGURE 6:**
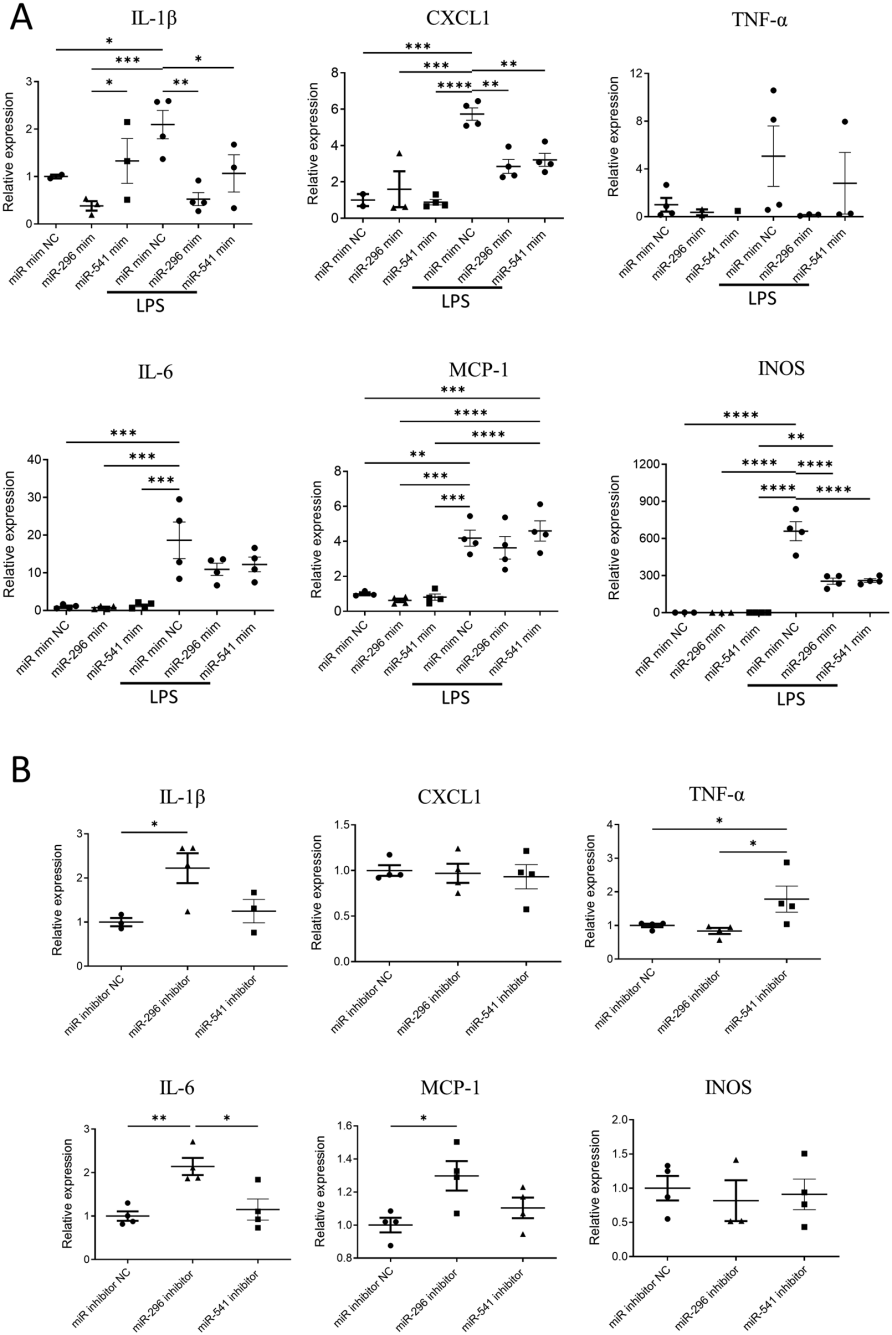
miR‐296‐5p and miR‐541‐5p reduce LPS‐induced inflammation. (A) MEF cells were transfected with miR‐296‐5p mimic or miR‐541‐5p mimic or negative control miRNA and incubated in the absence or presence of LPS. Cytokine expression was measured by real‐time PCR. Mean values are shown as fold induction relative to β‐actin and normalized to miRNA negative control. (B) MEF cells were transfected with miR‐296‐5p inhibitor or miR‐541‐5p inhibitor (antagomirs) and cytokine expression was measured by RT‐qPCR. Mean values are shown as fold induction relative to β‐actin and normalized to miRNA negative control. Data expressed as mean ± SEM; Statistical significance was defined as ****p<0.0001; ***p<0.001; **p < 0.01; *p < 0.05. *n* = 4. Representative of two independent experiments.

To further test whether the anti‐inflammatory effect observed with the young EVs were contributed by any of the two miRNAs identified, MEF cells were transfected with the miRNA mimics and treated with LPS. Although transfection of MEF with the miRNAs had an anti‐inflammatory effect, LPS treatment of the mimic transfected cells demonstrated a more profound inhibition of the inflammatory phenotype (Figure 6A). miR‐296‐5p or miR‐541‐5p mimics markedly reduced the gene expression of intracellular pro‐inflammatory cytokines such as *Il‐1β, Tnf‐α* and *Il‐6* and *Inos* in the MEF cells (Figure 6A). The anti‐inflammatory effect was not observed when MEF cells were transfected with antagomirs of miR‐296‐5p and miR‐541‐5p (Figure 6B). When either of the two miRNAs were inhibited with the antagomirs, the gene expression as assessed by real‐time PCR showed significant expression of several proinflammatory genes. Although miR‐296‐5p inhibition resulted in an increased expression of *Il‐1β, Il‐6, and Mcp1*, inhibition of miR541‐5p resulted in significant (over) expression of *Tnf‐α* demonstrating that both miR‐296‐5p and miR‐541‐5p possess anti‐inflammatory properties (Figure 6B).

### 3.7 Young EVs Containing miR‐296/541‐5p May Improve Wound Healing in PMVECs

**FIGURE 7:**
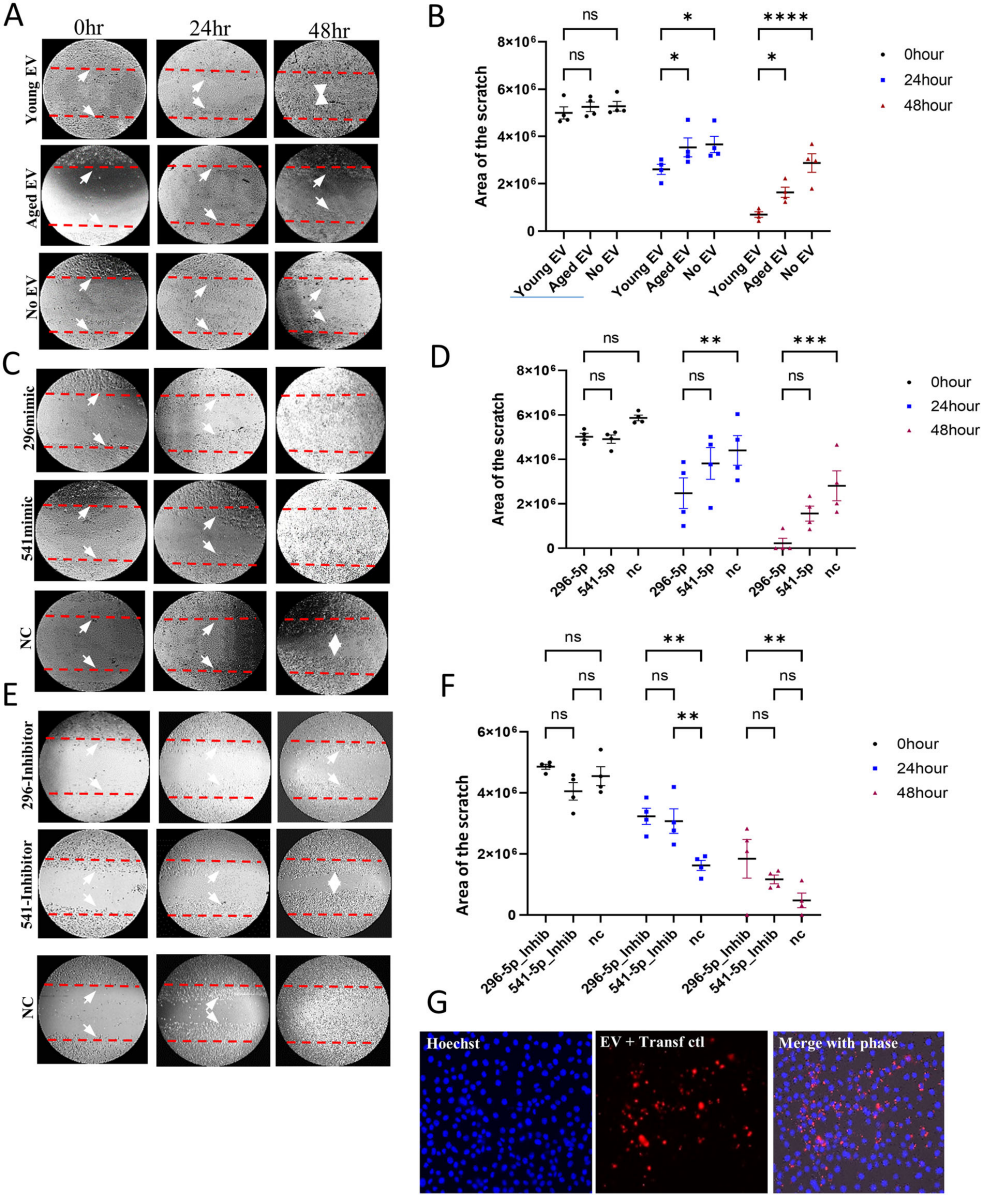
Young EVs, and miR‐296‐5p and miR‐541‐5p improve wound healing in an in vitro scratch assay model. (A) Rat PMVECs were plated, scratched and incubated with EVs isolated from young and aged mice at 0‐, 24‐, and 48‐h intervals. (B) Comparison of the area of scratch filled (migration) cells after treatment with young or aged EVs at different time points. (C) Rat PMVECs transfected with mimics of miR‐296‐5p or miR‐541‐5p, were plated, scratched, andobserved at 0‐, 24‐, and 48‐h intervals. (D) The area of scratch filled in cells at different time points after treatment with miR‐296‐5p or miR‐541‐5p mimics. (E) Rat PMVECs incubated with young EVs transfected with inhibitors of miR‐296‐5p and miR‐541‐5p, scratched and observed at 0‐, 24‐, and 48‐h intervals. (F) The change in the area of scratch after treatment with EVs transfected with antagomirs (inhibitor) of miR‐296‐5p and miR541‐5p at different time points. (G) Panels show EVs isolated from the plasma of young mice  transfected with a Cy‐3 labelled transfection control miRNA (red) using lipofectamine and incubated with the PMVECs. The nucleus was stained with Hoechst. Images were captured in the Echo Revolve microscope (Life Technologies) at 200× magnification. Graphical data shown as mean+SEM. Statistical significance expressed as * p < 0.05; ** p < 0.01; *** p < 0.001; **** p < 0.0001.

To compare and assess the regeneration induction potential of young and aged EVs and the two miRNAs as their functional component aiding in regeneration, we treated PMVECs with young and aged EVs or respective miRNAs in a scratch assay. The EVs derived from the plasma of young mice showed significant and faster healing of the scratched wound than those derived from aged mice in 48 h. Similarly, our experiments showed that when the cells were treated with miR‐296‐5p or miR‐541‐5p mimics the scratched surface showed significantly higher wound closing compared to that treated with negative control. Furthermore, the inhibition of miR‐296‐5p by the antagomir was more effective in slowing the wound healing process in comparison to miR‐541‐5p antagomir by 48 h (Figure 7A–G).

### 3.8 miR‐296‐5p Rescues Mice From CLP‐Induced Sepsis

**FIGURE 8:**
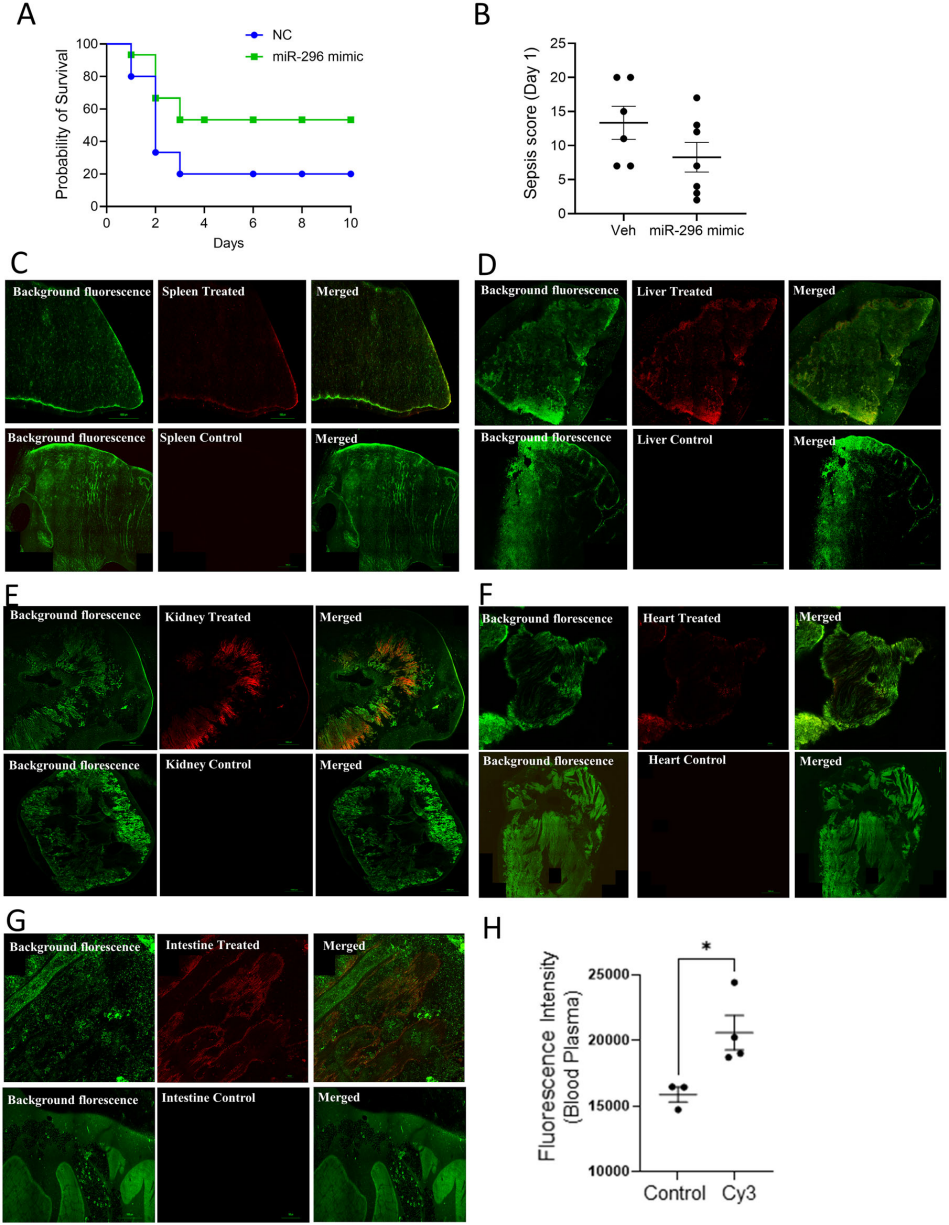
miR‐296‐5p improves survival following CLP‐induced sepsis. (A) Kaplan–Meier survival curves of mice subjected to CLP‐induced sepsis and treated (i.p.) with miR‐296‐5p negative control (NC) (*n* = 15) or miR‐296‐5p mimic (*n* = 15). Composite data from three independent experiments. Veh. versus miR‐296‐5p, *p* = 0.046. (B) Twenty‐four hours after CLP surgery, the sepsis score showed less severity in the group treated with miR‐296‐5p ‐. (C–G) Confocal large, stitched image snapshots of the murine spleen, liver, kidney, heart and intestine showing biodistribution of Cy3‐labeled non‐targeting miRNA injected i.p as in the experiment shown in panel A above. Images were captured from whole mount longitudinal section of tissues removed 3 h after i.p. injection. Cy3‐non‐targeting control miRNA (red) was observed in a whole mount tissue over a dull green autofluorescence in the spleen, liver, kidney, heart, and intestine. No red fluorescence was observed in organs of control mice. Green autofluorescence images allow tissue visualization. All images were captured with a Nikon–Nsparc inverted confocal microscope with a ×20 Plan Apo objective lens. (H) The graph shows a significant increase in fluorescence intensity in the plasma of mice at 3 h after injecting (i.p.) with Cy3 non‐targeting miRNA compared to the plasma from control mice. Graphical data represented as mean+SEM. *p<0.05.

Our next goal was to determine whether miR‐296‐5p and miR‐541‐5p can rescue mice from death after CLP surgery. We delivered miR‐296‐5p, miR‐541‐5p or control miRNA intraperitoneally 24 h before and 2 h after the CLP surgery in each mouse. The preliminary experiments with five mice showed similar effects for miR‐541‐5p and the control miRNA. We further investigated the effect of miR‐296‐5p on sepsis‐induced mortality. Mice were monitored for 10 days and the miR‐296‐5p recipient mice showed significantly improved sepsis score and survival (Figure 8A,B). We also show that the miRNA injected by i.p. route reaches plasma (Figure 8H) and several tissues (Figure 8C–G) within 3 h. To further confirm the role of miR‐296‐5p in the inflammatory response, we performed RNAseq of MEF cells treated with LPS with and without exogenously supplemented microRNA and found that miR‐296‐5p reduced the expression level of 222 genes while upregulated only 32 genes (> or <two‐fold and *p *< 0.05; data not shown). miR‐296‐5p reduced the expression of inflammation‐associated genes such as *Cxcl10, Cxcl11, Mpeg1* and *Pax5*. These results further demonstrate an anti‐inflammatory effect for miR‐296‐5p.

## 4 Discussion

Aging systems gradually lose agility, with a decline in physiological function (Jian et al. 2011; Khan et al. 2017; Reitsema et al. 2024; Guo et al. 2022). This deceleration of growth and acceleration of aging begins early in life, as it has been shown that age‐associated changes in gene expression originate during the juvenile period of growth deceleration (Lui et al. 2010). Furthermore, the susceptibility to injury‐induced death increases with age (Jian et al. 2011; Harris et al. 2003; Wongweerakit et al. 2022; Jian et al. 2011). In this report, for the first time, we show that when sepsis was induced in mice ages 5 weeks to 2 years, the mice at or before puberty were more resilient to the adverse sequel following the CLP surgery. Although all the mice in the aged group died within 3 days, half of the mice in the 5–6‐week‐old group survived after CLP. Though the mean survival duration of mature (14–16‐week‐old) mice was longer than that of the aged mice, it was significantly shorter than that of the young mice. This experiment demonstrates that very young animals are more resilient in responding to injury, infection and stress. However, the causative factors for the resiliency of the young mice are unknown, and the factors in the young blood responsible for the reparative effect in the aged systems also remain unknown.

Recently, some reports have defined proteins such as platelet factor 4 (PF4) and KLOTHO as rejuvenating factors. In one of the studies, when aged male mice were systemically treated with young blood plasma or platelet fraction, profound gene expression changes associated with immune regulation and nervous system development were observed in the brain; however, these changes were not observed with blood plasma or platelet fraction from the aged mice (Schroer et al. 2023). Systemic injection of PF4, which was found to be elevated in the young plasma, into aged mice reduced inflammation in the hippocampus and improved cognition (Schroer et al. 2023). Simultaneously, another group found that systemic increase in PF4 improved age‐related cognitive impairments and hippocampal neurogenesis (Leiter et al. 2023). Furthermore, the same group showed that exercise activates platelets that are essential for exercise‐induced neurogenic processes. In a different study, when mice were treated with PF4, synaptic plasticity and cognitive function were improved in the young mice and PF4 reduced cognitive defects in the aged brain (Park et al. 2023). Though they observed similar improvement with KLOTHO, KLOTHO‐mediated cognitive enhancement was abrogated by inhibiting platelet activation, speculating PF4 or yet other mediators in the young blood to be the effector (Park et al. 2023). The presence of KLOTHO in EVs from young serum was previously found to regulate skeletal muscle regeneration in the aged mice (Sahu et al. 2021). EVs from the young blood were a focus of similar studies which showed that the young EVs exhibited GSTM2 activity, reversed oxidative stress and reduced senescence‐associated tissue damage (Chu et al. 2022; Fafian‐Labora et al. 2020).

We recently demonstrated that intravenous administration of EVs derived from the plasma of mice at or before puberty (less than 2‐month‐old) can improve survival following haemorrhagic shock, an acute injury (Chu et al. 2022). However, the identity of the molecular entities in the cargo of the EVs that imparted the reparative effect remained unknown. The present report shows that EVs isolated from 5–6‐week‐old (young) mice improved outcomes following sepsis induced by CLP surgery. The EVs are rich in exosomes, as shown by the presence of exosomal markers by Western blot and immunoelectron microscopy. Using SPECT/CT imaging, we show that the EVs injected into the tail vein home to several internal organs, ruling out that the effect is restricted to any single organ.

Our experiments show that when mice were injected with EVs derived from blood plasma, the donor age of the EVs is a critical factor in determining the outcome after sepsis. The mortality after sepsis induction was the lowest among the recipients when the EV donors were at or before pubertal age. The EVs from the young mice reduced cytotoxicity, suppressed senescence markers and downregulated inflammatory genes in MEF cells treated with LPS. The reduced level of mtDNA in the young plasma EVs also supports their reduced inflammatory properties when compared to EVs from the aged mice. Our scratch wound experiments using rat PMVECs show that young EVs can improve cellular regeneration in comparison to EVs from aged mice.

To determine the molecular identities of young plasma factor(s) contributing to improved survival after sepsis, we analysed the miRNA content of the EV cargo. Although a number of miRNAs were identified in the EVs, two miRNAs showed a progressive decline with age, miR‐296‐5p and miR‐541‐5p. Interestingly enough, both miRNAs are expressed in most tissues, with a sharp decline in expression with progressing age, or more specifically post maturation. Although both miRNAs demonstrated anti‐inflammatory effect, the antagomirs of both miR‐296‐5p and miR‐541‐5p were able to induce significant expression of inflammatory cytokines demonstrating that miR‐296‐5p may be a potent inhibitor of inflammation. The lack of a similar increase of inflammatory cytokines in MEF cells treated with the mimics of the two miRNAs and the ability of the mimics to suppress LPS‐induced inflammation in MEF cells further support the inference that miR‐296‐5p and miR‐541‐5p have anti‐inflammatory‐effects, miR‐296‐5p being more effective.

Our experiments also show that the presence of miR‐296‐5p and miR‐541‐5p mimics may aid the regeneration process by the young EVs. Nevertheless, the inhibition of both miRNAs decelerated the wound healing in the scratch assay. A recent study showed that transplanting aged human skin onto 2‐month‐old young SCID/beige mice rejuvenated the xenotransplants. However, when the same was transplanted to 14‐month‐old mice, no rejuvenation effect was observed in the old human skin (Keren et al. 2022). The rejuvenation effect was followed by a significant increase in VEGF expression in the young, while the rejuvenation effect of the recipient mouse was lost with progressing age. Furthermore, VEGF has been previously proposed to be a key driver of human organ rejuvenation and its level was found to be reduced with age (Keren et al. 2022). On the contrary, VEGF promotes vascular permeability, an important pathophysiological mechanism of sepsis, with a positive correlation between VEGF levels and severity/mortality (Tang et al. 2022). Importantly, the VEGF has been previously shown to increase the expression of miR‐296, also termed by some as angiomiR. miR‐296 was reported to be elevated during angiogenesis and reduced the substrate that degrades VEGFR2 and PDGFR‐β promoting angiogenesis (Wagatsuma 2006; Anand and Cheresh 2011; Wurdinger et al. 2008). The inhibition of miR‐296 abolished VEGF‐induced angiogenesis, suggesting this miRNA's role in angiogenesis and rejuvenation. However, our studies indicate that neither miR‐296‐5p nor miR‐541‐5p upregulates VEGF. Instead, we found a downregulation of VEGF when MEF cells were treated with the respective mimic in the absence or the presence of LPS (data not shown, RNA seq).

Our observation that i.p. treatment of mice with miR‐296‐5p reduced mortality following sepsis suggests a critical role for miR‐296‐5p target in sepsis pathogenesis. The transport and uptake of miRNA from the peritoneal cavity to the systemic circulation and other organs were facilitated by using the transfection agent ‘in vivo‐JetPEI,’ which has been successfully used in several in vivo models (Takao et al. 2020). The intraperitoneally administered agents diffuse into the surrounding tissues (as shown using Cy3 labelled control miRNA), enter the blood and lymph vessels, and exert an anti‐inflammatory effect. Furthermore, our in vitro studies confirm an anti‐inflammatory effect for the micro RNAs, though the specific mechanism remains to be understood. Another significant advantage of intraperitoneal administration is slow absorption into systemic blood circulation (Turner et al. 2011). Similar strategies were adopted by others with success (Fafian‐Labora et al. 2020; Dragomir et al. 2023). After all, systemic inflammation in experimental sepsis has its origin in the infectious foci in the peritoneum.

One of the limitations of the study is that, in the LPS transfection experiments presented, the miRNAs were transfected into the cells and induction with LPS was followed by the transfection. However, it may be noted that in vivo, the cells would be affected by sepsis prior to miRNA treatment. Furthermore, the uptake of the EVs by the cells and the effect of the miRNA after uptake are also important factors to be taken into consideration. Nevertheless, in the scratch assay, we treated the cell monolayer with young and old EVs after scratching the monolayer, demonstrating its therapeutic potential. Another limitation is that both male and female mice could have been used to address the effect of sex as a biological variable.

In summary, we demonstrate that EVs from very young mice have a reparative effect on wound healing and sepsis, and the reparative factors are likely maturation‐dependent. Our observation that miR‐296‐5p is a plasma EV constituent that significantly reduces with age and can reduce mortality following sepsis suggests the therapeutic potential for this miRNA in sepsis and possibly aging and age‐associated co‐morbidities.

## Author Contributions

**Lun Cai**: Formal analysis (equal), investigation (equal), methodology (equal), writing – original draft (equal). **Parmita Kar**: Investigation (equal), methodology (equal), writing – review and editing (equal). **Yutao Liu**: Data curation (equal), investigation (equal), methodology (equal), resources (equal). **Xiaogang Chu**: Investigation (equal), methodology (equal). **Ashok Sharma**: Data curation (equal), formal analysis (equal), methodology (equal), writing – review and editing (equal). **Tae Jin Lee**: Data curation (equal), formal analysis (equal), methodology (equal), software (equal), validation (equal), writing – review and editing (equal). **Ali Arbab**: Data curation (equal), formal analysis (equal), resources (equal), writing – review and editing (equal). **Raghavan Pillai Raju**: Conceptualization (equal), investigation (equal), methodology (equal), resources (equal), supervision (equal), validation (equal), writing – original draft (equal).

## Conflicts of Interest

The authors declare no conflicts of interest.

## Data Availability

Contact the corresponding author for source data not provided with the manuscript. Genomics data will be uploaded to a public server upon acceptance of the paper.
